# An Alarmingly High Number of Candidates for Bariatric Procedures among Professionally Active Poles and Its Strong Relationship with Cardiovascular Co-Morbidities—POL-O-CARIA 2022 Study

**DOI:** 10.3390/jcm12206431

**Published:** 2023-10-10

**Authors:** Anna Rulkiewicz, Iwona Pilchowska, Wojciech Lisik, Piotr Pruszczyk, Simona Wójcik, Marcin Poboży, Justyna Domienik-Karłowicz

**Affiliations:** 1 LUX MED, Postępu 21C, 02-676 Warsaw, Polandsimona.wojcik@luxmed.pl (S.W.); 2 Department of Psychology, SWPS University of Social Sciences and Humanities, 03-815 Warsaw, Poland; 3 Department of General and Transplantation Surgery, Medical University of Warsaw, 02-005 Warsaw, Poland; wojciech.lisik@wum.edu.pl; 4 Department of Internal Medicine and Cardiology, Medical University of Warsaw, 02-097 Warsaw, Poland; piotr.pruszczyk@wum.edu.pl; 5 HealthCare Facility Cichowski-Poboży, 08-480 Maciejowice, Poland; marcinpobozy1@gmail.com

**Keywords:** BMI (kg/m^2^), professionally active adult population, cardiovascular diseases, obesity, bariatric procedures

## Abstract

Over recent years, the global healthcare system has experienced a notable increase in the prevalence of obesity and its associated health complications such as hypertension, type 2 diabetes, lipid disorders, etc. What is more, one of the significant phenomena is the increasing demand for bariatric procedures among individuals of working age due to the high prevalence of type III obesity and type II obesity with co-morbidities. This trend is pronounced in Poland, due to the increasing number of patients meeting the qualifying criteria for surgery among professionally active and inactive patients. The aim of this study is to characterize the alarmingly high number of candidates for bariatric procedures among professionally active Poles. In total, the results of 2,056,861 initial, control, and periodic visits as part of the occupational medicine certificate were analyzed—collected from 1,342,749 unique patients (51.7% men; mean age of whole group: 36.81, SD = 10.91). Statistical calculations were performed, qualitative data were assessed using percentage and occurrence counts, while qualitative data were described using mean (M), standard deviation (SD), median, skewness, kurtosis, and range values. Results with *p* < 0.05 were deemed significant. Chi-square analysis and one-way ANOVA (with Scheffe’s post hoc test) were employed. Charts were created in the R program. It was noticed that there was a consistent rise in the proportion of individuals classified as candidates for bariatric procedures (an increase of 0.3%) alongside a notable decrease in the percentage of individuals maintaining a healthy body weight. Moreover, it is imperative to conduct yearly evaluations of the prevalence of obesity and its associated health conditions. It should be noted that hypertension occurred in 42.2% of patients, type 2 diabetes in 6.2% of patients, and lipid disorders in 8.4% of patients with third-degree obesity. This proactive approach is essential in order to adequately equip the healthcare system to address the increasing population of obese individuals, especially candidates for bariatric procedures.

## 1. Introduction

In recent years, the global healthcare system has observed a notable increase in the prevalence of obesity and its associated health complications [1,2]. Furthermore, a rising demand for bariatric procedures among working-age individuals has emerged as a noteworthy phenomenon [3]. This trend is especially pronounced in Poland, due to the growing number of professionally active patients meeting the surgical qualifying criteria. This study examines the concerning increase in professionally active Poles seeking bariatric interventions and explores the strong correlation between this trend and cardiovascular co-morbidities.

It is worth noting that the escalating prevalence of obesity has raised alarms not just regarding individual health, but also concerning the strain on healthcare systems and global economies [4]. Bariatric procedures have emerged as crucial interventions in addressing severe obesity and its related health risks, offering the potential for improved quality of life and long-term health outcomes [5,6,7]. However, the specific implications of this surge, particularly among those in professional roles, warrant further investigation. The POL-O-CARIA 2022 study focuses on the rising inclination among professionally active Poles towards bariatric procedures. By examining the main factor driving this trend and the cardiovascular co-morbidities commonly associated with it, the study aims to contribute valuable insights to both medical practitioners and policymakers. Recognizing the intricate association between the surging demand for bariatric surgeries and cardiovascular health can guide specialized interventions, public health initiatives, and policy strategies to alleviate the health and economic repercussions of obesity-linked diseases [8].

Through a comprehensive analysis of data and trends, the POL-O-CARIA 2022 study seeks to provide a comprehensive understanding of the complex interplay between bariatric procedures, cardiovascular co-morbidities, and the professional lives of individuals in Poland. This research has the potential to drive informed decision-making in healthcare, facilitating proactive strategies that address the multifaceted challenges posed by the increasing prevalence of obesity and its associated health consequences among the working-age population.

### 1.1. Obesity and Co-Morbidities

Beyond its evident impact on physical appearance, obesity is closely associated with a spectrum of accompanying health conditions that significantly compromise individual well-being. Numerous research studies documented in existing literature underscore the strong link connecting excess weight and obesity to the onset of a diverse range of medical conditions [9]. A comprehensive meta-analysis conducted in 2015 yielded illuminating insights, revealing that even a moderate 5 kg increase in weight substantially increases the risk of certain cancers. The analysis specifically showed an 11% heightened risk for post-menopausal breast cancer, a 39% surge in the risk of endometrial cancer, a 13% rise in the risk of ovarian cancer, and a 9% growth in the risk of colon cancer for men [10]. Further explorations through cohort investigations, undertaken as part of the Me-Can 2.0 program across several European nations such as Austria, Norway, and Sweden, corroborated these findings. These investigations illuminated that individuals classified as overweight up until the age of 40 exhibit a significant rise in the probability of developing various malignancies. Notably, these encompassed increased risks for endometrial cancer, renal-cell cancer and colon cancer in men [11]. Beyond its immediate impacts, obesity assumes the status of a chronic metabolic disorder that profoundly influences the prevalence of cardiovascular diseases. Excessive fat accumulation impacts cardiac function directly by influencing the myocardium and blood vessels, and indirectly through associated health issues. The building up of too much fat tissue causes circulatory changes, such as increased blood volume and heart activity along with decreased overall blood vessel resistance. This weight gain is also tied to increased blood pressure, triggered by the renin–angiotensin–aldosterone and sympathetic nervous systems. Furthermore, obesity directly harms the heart muscle through fat buildup leading to subsequent scarring, potentially resulting in LVDD and HF with a preserved ejection fraction (HFpEF) [12]. Comprehensive profiling of HFpEF patients, both with and without obesity, in contrast to normal individuals, revealed that obese individuals with HFpEF exhibited pronounced LV adjustments, enlargement of the right ventricle, and associated dysfunction. Scientific research leaves little room for doubt regarding the complex connections between obesity and conditions such as hypertension, coronary artery disease, atrial fibrillation, obstructive sleep apnea, non-alcoholic fatty liver disease, and diabetes [2,9,12,13,14]. The body of evidence not only underscores the complexities of obesity’s impacts on health but also emphasizes the urgency of comprehensive approaches to address its wide-ranging consequences [2]. First of all, we should underline who is eligible for bariatric surgery. Above all, this should include individuals who have not achieved weight loss through diet, physical activity, and pharmacotherapy, with a body mass index (BMI) of 40 or more kg/m^2^, indicating severe obesity. Moreover, those with a BMI ranging from 35 to 39.9 kg/m^2^ (considered obese), along with a significant weight-related medical issue like type 2 diabetes, hypertension, or severe sleep apnea should also be included.

### 1.2. Obesity and Professional Activity

The impact of work on overweightness and obesity has been acknowledged. Employers take steps to encourage healthy eating habits and boost physical activity among their staff. Providing complimentary fruits and vegetables at workplaces is becoming more common. Factors within the work environment that can contribute to overweightness and obesity encompass sedentary job roles, stress, and sleep issues [15]. Engaging in office work and sedentary tasks escalates the susceptibility to obesity among employees. A study by Shields and Tremblay in 2008 affirmed a positive correlation between obesity and prolonged sitting during leisure time, such as when using a computer [15]. Conversely, there are various studies that refute the link between sedentary work or leisure pursuits and the prevalence of overweightness and obesity. Stress encountered in the workplace is also a significant factor associated with overweightness and obesity.

## 2. Aim of the Study

The aim of this study is to characterize the alarmingly increasing number of candidates for bariatric procedures among professionally active Poles who underwent occupational medicine examinations in Poland in 2016–2022 and its strong relationship with cardiovascular co-morbidities (POL-O-CARIA 2022 study). Because the study aims to explore rather than confirm specific research hypotheses, the article does not propose any research hypotheses. Instead, we formulated research inquiries that delineated the primary focus of the investigations: examining how the severity of obesity evolves over time and its interactions with other medical conditions.

## 3. Materials and Methods

This paper examines the findings of the POL-O-CARIA 2016–2022 study, focusing on professionally active adults seen from January 2016 to September 2022 for occupational health purposes. The LUX MED Group supplied the data. In all, 2,056,861 initial, control, and periodic occupational health check-ups were reviewed. They were collected from 1,342,749 unique patients (51.7% men; mean age of whole group: 36.81, SD = 10.91). Throughout the research, factors such as gender, age, residency province, medical certification period, and medical history elements like self-assessed health and smoking habits were monitored. Over the years, there has been a steady rise in the percentage of individuals categorized as overweight or obese. This makes it crucial to regularly monitor obesity prevalence in different societal groups. Studying the health of working Polish adults is important for multiple reasons, including monitoring overall health and anticipating the onset of certain lifestyle-related diseases in specific groups. The existence of conditions like obesity, hypertension, type 2 diabetes, sleep apnea, and non-alcoholic steatohepatitis often results in fewer medical approvals for job roles.

Obesity is generally classified based on body mass index (BMI), which is a numerical value derived from an individual’s weight and height. While the exact thresholds can vary slightly depending on the source or country, the World Health Organization (WHO) and many other health institutions use the following classifications for adult obesity:

1. Class 1 Obesity: A BMI of 30.0 to 34.9.
2. Class 2 Obesity: A BMI of 35.0 to 39.9.
3. Class 3 Obesity: A BMI of 40.0 and above.

The European Society of Hypertension (ESH) and the European Society of Cardiology (ESC) provide guidelines on the management of arterial hypertension. According to the ESH/ESC, arterial hypertension is usually defined as a systolic blood pressure (SBP) of 140 mmHg or higher, and/or a diastolic blood pressure (DBP) of 90 mmHg or higher. For the diagnosis of type 2 diabetes, we use the general criteria, which typically include:

1. Fasting Plasma Glucose: ≥7.0 mmol/L (or ≥126 mg/dL).
2. 2 h Plasma Glucose during an Oral Glucose Tolerance Test (OGTT) with 75 g of glucose: ≥11.1 mmol/L (or ≥200 mg/dL).
3. HbA1c (Glycated Hemoglobin): ≥6.5%.
4. Random Plasma Glucose: ≥11.1 mmol/L (or ≥200 mg/dL) in the presence of diabetes symptoms.

### Statistical Analysis

Statistical analyses were conducted utilizing IBM SPSS Statistics 25. For the examination of qualitative data, we utilized percentages and frequency counts. To describe qualitative data, we relied on metrics such as mean (M), standard deviation (SD), median, skewness, kurtosis, as well as the smallest and largest values. Results were deemed statistically significant if the likelihood of a type I error was below 5% (*p* < 0.05). For our statistical evaluations, we employed chi-square tests in contingency tables (with Bonferroni correction applied for assessing column ratios) and a one-way ANOVA (utilizing Scheffe’s post hoc test for contrasting means). Graphical representations were crafted using the R software 4.2.3.

## 4. Results

### 4.1. Information on BMI (kg/m^2^)

It was noticed that, over successive years of observation, there was a consistent rise in the proportion of individuals classified as overweight or obese (regardless of the level of severity), alongside a notable decrease in the percentage of individuals maintaining a healthy body weight. Detailed results are presented in Table 1.

In relation to body mass index (BMI), it was discovered that individuals with a normal body weight, who were actively engaged in their professions, obtained the longest medical certificates. These certificates lasted for approximately 34 months. For those categorized as overweight or dealing with obesity, a clear correlation emerged between the severity of obesity and the duration of medical certification (refer to Table 2). Based on medical decisions, individuals with overweight conditions were granted work-related medical certificates, averaging around 31 months. The capacity to remain occupationally active notably declined as the obesity level escalated: 1st degree obesity led to an average certificate duration of about 28 months, 2nd degree obesity was associated with roughly 27 months, and 3rd degree obesity resulted in nearly 26 months of certification—we present these details in Table 2.

### 4.2. Patient Characteristics Depending on BMI (kg/m^2^) Level

Chi-square analysis showed that similar trends were observed for both women and men regarding the dynamics of occurrence of individual BMI (kg/m^2^) categories. In both groups, a noteworthy decline was identified on an annual basis among individuals with a normal body weight. Additionally, the inclination towards an increase in individuals categorized with 1st and 3rd degree obesity was more pronounced among men. Here, we observed a concerning rise in potential candidates for bariatric surgery (see Table 3).

**Figure 1:**
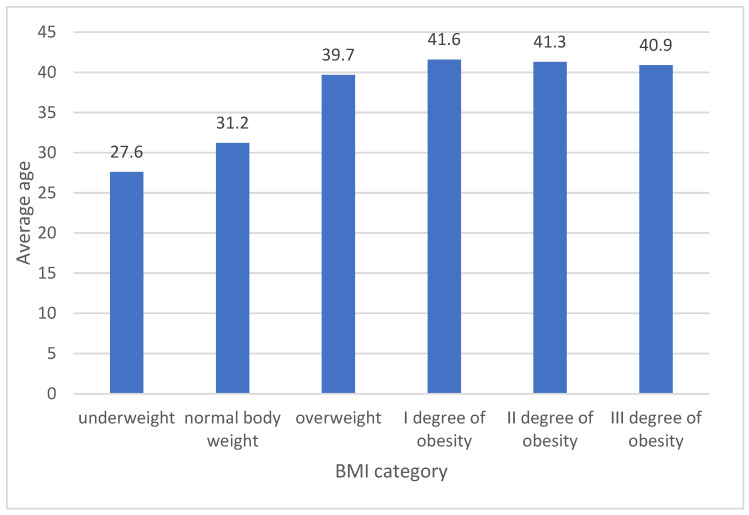
The average age varies based on BMI category (kg/m^2^). In the graph, all group differences are statistically significant at a *p* < 0.001 threshold. Due to the multitude of groups being compared, specific differences are not displayed in the figure.

Notable disparities were observed in a one-way variance analysis for age and the duration for which measurement were taken, irrespective of the measurement year (in both instances, the variation between groups was significant at *p* < 0.001). More detailed results are elaborated on below. Regarding the age of the patients, a post-hoc examination using Scheffe’s adjustment indicated that only between individuals with II and III obesity degrees were there no age differences. In other comparisons, the significance of variations between specific groups stood at *p* < 0.001. The oldest average age was noted among those with obesity, whereas the youngest was among individuals with underweight or standard weight (see Figure 1).

Individuals with a standard weight were predominantly found in the under 35 age group. Conversely, the proportion of those with obesity, particularly of the 1st degree, rose notably across all age groups (refer to Table 4).

Table 5 displays the data by adjusting the percentages based on BMI categories. The findings indicate that as BMI increases, the proportion of individuals under 35 years of age diminishes in every group. For those aged between 35 and 69, it was observed that they were more frequently categorized into the overweight or obesity groups compared to those with standard weight.

**Figure 2:**
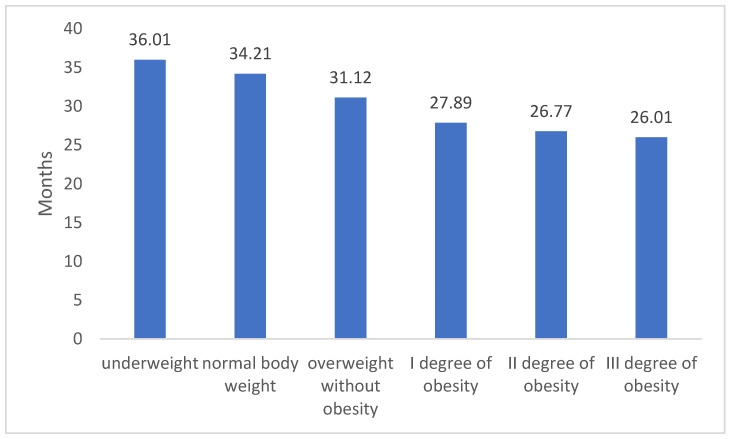
The mean duration in months of the provided medical certificate varies based on BMI category. In the chart, every group showcases notable statistical differences at a *p* < 0.05 threshold. Owing to the extensive group comparisons, specific distinctions are not illustrated in the figure.

In the examination of the durations for medical certificate issuance, notable variations among the groups were identified. A linear trend was evident, demonstrating that as the BMI level rises, the average duration of the issued certification shortens- what we present on Figure 2. Furthermore, a post-hoc evaluation using Scheffe’s adjustment highlighted significant disparities across all BMI categories. More comprehensive outcomes are delineated in the subsequent sections. 

**Figure 3:**
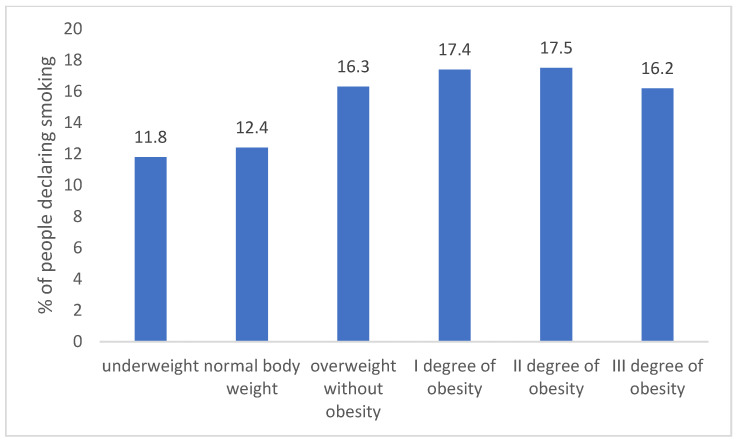
Percentage of people declaring smoking depending on BMI category (due to the number of groups compared, results for differences are not shown in the figure).

Individuals with standard or underweight showed a lower tendency to smoke compared to those who were overweight or obese. This distinction is statistically significant at a minimum threshold of *p* < 0.05. Such a pattern was consistent across all measurement years, as depicted in Figure 3.

An exploration was conducted into the association between BMI classifications and individuals’ self-evaluation of health. It was observed that those who rated their health as “good” were less frequently categorized as having normal weight compared to those who rated their health as “very good”. The opposite trend was evident for individuals who were overweight or obese. More in-depth findings are elaborated on in the following sections and in Table 6.

Table 7 illustrates the connection between chosen ailments and BMI classifications. A notable association between these variables was determined (*p* < 0.001). The most distinct variations were seen in hypertension (as BMI increased, the proportion of individuals with this condition rose) and in lipid imbalances and type 2 diabetes.

### 4.3. BMI and Observed Co-Morbidities

A notable correlation was found between BMI (kg/m^2^) classifications and the presence of co-morbidities (chi2 (70) = 16138; *p* < 0.001). In-depth findings indicated that among patients diagnosed with hypertension or lipid imbalances, there were significant variations across all groups. Specifically, as BMI escalated, the likelihood of each condition’s presence also grew. It should be noted that hypertension occurred in 56.1% of patients, type 2 diabetes in 17.1% of patients, and lipid disorders in 24.1% of patients with third-degree obesity. A Table 8, further down provides a comparative overview of all co-morbidities based on BMI levels.

It should be noted that hypertension occurred in 42.2% of patients, type 2 diabetes in 6.2% of patients, and lipid disorders in 8.4% of patients with third-degree obesity. The cross-tabulation chi-square analysis performed confirmed that there was an association between age and co-morbidities (chi2(56) = 27809.28; *p* < 0.001). In the case of hypertension, it was obtained that the prevalence of hypertension was more common in those aged 18–54 compared to other age groups. In addition, the prevalence of lipid disorders was significantly different in each of the age groups—a trend was observed showing that the diagnosis of this disease decreased with age. A detailed comparison of the age groups for the other diseases is shown below, in Table 9.

## 5. Discussion

In this research study, we utilized data obtained from a substantial cohort of 1,450,455 distinct adult patients. Employing a comprehensive analytical approach, we derived estimations regarding the trends in body mass index (BMI); while previous analyses have explored the prevalence of obesity in Poland in preceding years, none have focused on the present time frame nor have they encompassed such an extensive patient pool [16].

A distinctive aspect of our analysis lies in its incorporation of correlations between BMI and critical factors, including the average duration of medical certificates issued and the concurrent presence of severe illnesses primarily associated with the cardiovascular system. Notably concerning is the revelation that as BMI escalates, individuals’ capacity to remain productive within the workforce diminishes. Importantly, our study does not encompass individuals who, due to obesity and its related co-morbidities, are unable to engage in work altogether.

It is paramount to emphasize that, within this vast patient cohort, we have established the coexistence of ailments that significantly affect patients’ overall quality of life [7]. The extent of such co-morbidities distinctly corresponds to the degree of obesity. Our data unequivocally demonstrated that approximately one third of active female professionals and nearly two thirds of their male counterparts fall within the overweight or obese categories. This finding is deeply disconcerting.

Morbid obesity is increasingly recognized as a pivotal factor in the development and progression of several co-morbid conditions, notably hypertension, type 2 diabetes, and dyslipidemia. Numerous studies have elucidated the intricate pathways through which excessive adipose tissue can drive the onset of high blood pressure. Mechanisms such as increased renal sodium retention and heightened sympathetic nervous system activity have been identified as mediators of this relationship. Furthermore, the secretion of adipokines from visceral fat, abundant in morbidly obese individuals, can lead to systemic inflammation and impaired insulin signaling, setting the stage for the onset of type 2 diabetes mellitus. The elevated risk of developing this form of diabetes is particularly pronounced in those with extreme levels of obesity. Concurrently, the perturbed lipid metabolism in these individuals gives rise to dyslipidemia, characterized by imbalances in LDL cholesterol, triglycerides, and HDL cholesterol levels. The simultaneous presence of these conditions in morbidly obese individuals amplifies their risk of cardiovascular diseases. It is noteworthy that weight loss interventions, especially bariatric surgery, have shown remarkable efficacy in resolving or significantly improving these co-morbidities. Additionally, the economic ramifications of these interconnected conditions emphasize the importance of early and holistic interventions. Contemporary research advocates for a collaborative approach to patient care, drawing on the expertise of cardiologists, endocrinologists, and obesity specialists. Addressing the root cause, morbid obesity, can have cascading benefits, potentially reversing the associated co-morbidities and enhancing the overall quality of life for affected individuals. The interplay of these conditions underscores the multifaceted challenges posed by morbid obesity and necessitates comprehensive, evidence-based strategies for effective management.

Furthermore, our study illuminates an alarming temporal trend that indicates a surge in this phenomenon over time. This development raises apprehensions regarding both access to medical services and the economic burden on the healthcare system. The data underscore the non-uniform distribution of this issue across various regions of the country. Appendix A provide unique insights delineating how obesity diversifies based on geographic regions.

Whereas grade II and grade III obesity were once exceptional occurrences, our findings indicate that they may soon dominate the spectrum of BMI categories within patient populations. Given the existing limitations in physicians’ capacity to effectively manage obese patients, the persistent progression of this trend is poised to become a formidable challenge for the entirety of the healthcare sector.

Bariatric surgery in particular has emerged as a game-changing intervention for patients with morbid obesity and those with grade II obesity accompanied by co-morbid conditions. The transformative potential of such procedures extends beyond mere weight reduction. Studies have consistently demonstrated the ability of bariatric interventions, like gastric bypass or sleeve gastrectomy, to induce remission of type 2 diabetes, often independent of significant weight loss. This remarkable effect is believed to be mediated through hormonal changes, improvements in insulin sensitivity, and alterations in gut microbiota.

Moreover, bariatric surgery has been found to induce significant and sustained reductions in blood pressure, thereby directly addressing hypertension in this patient population. Patients post-surgery often experience a favorable shift in their lipid profile, with decreases in LDL cholesterol and triglycerides and an elevation in protective HDL cholesterol. This reconfiguration of the lipid landscape contributes to the reduction in cardiovascular risks associated with morbid obesity.

The long-term benefits of bariatric surgery also include a reduction in the mortality rates from cardiovascular events and certain cancers. Economic evaluations reveal that, while the upfront costs of bariatric procedures are significant, the resultant savings from reduced medication requirements, fewer hospitalizations, and diminished need for interventions related to co-morbidities, make it cost-effective in the long run.

However, as transformative as bariatric surgery can be, it is vital to recognize its role as part of a comprehensive care package. Nutritional guidance, psychological support, and regular medical follow-ups are essential components that ensure the success and sustainability of surgical interventions. An individualized, patient-centric approach, where surgery is complemented by lifestyle and behavioral modifications, offers the best chance of restoring health and vitality to those grappling with the multifarious challenges of type II and III obesity.

## 6. Conclusions

In addition to the above, is imperative to conduct yearly evaluations of the prevalence of obesity and its associated health conditions. This proactive approach is essential in order to adequately equip the healthcare system for addressing the increasing population of obese individuals—candidates for bariatric procedures, who are actively engaged in their professions. By doing so, we can strategically implement the most efficient interventions to counteract this trend and ensure optimal health outcomes.

## Figures and Tables

**Table 1:** BMI (kg/m^2^) distribution depending on the year of measurement.

	2016	2017	2018	2019	2020	2021	2022	Total
Underweight	3.4%	3.4%	3.4%	3.2%	3.1%	3.0%	2.8%	3.0%
Normal body weight	51.6%	51.1%	50.1%	49.3%	48.2%	48.1%	47.6%	49.3%
Overweight	31.4%	31.5%	32.0%	32.2%	32.1%	32.4%	32.7%	32.4%
I degree of obesity	10.4%	10.7%	11.0%	11.5%	12.1%	12.3%	12.4%	12.4%
II degree of obesity	2.5%	2.6%	2.7%	2.9%	3.7%	4.1%	4.4%	2.6%
III degree of obesity	0.7%	0.7%	0.8%	0.8%	0.8%	0.1%	0.1%	0.3%
Total	100.0%	100.0%	100.0%	99.9%	100.0%	100.0%	100.0%	100.0%

**Table 2:** Descriptive statistics on the average number of months of the issued medical certificates depending on BMI (kg/m^2^).

	Underweight	Normal Body Weight	Overweight without Obesity	I Degree of Obesity	II Degree of Obesity	III Degree of Obesity
M	36.01	34.21	31.12	27.89	26.77	26.01
Me	36.00	36.00	36.00	23.00	23.00	23.00
SD	11.67	12.83	13.78	15.23	14.29	13.71
Skewness	−0.54	−0.62	−0.34	−0.14	0.23	0.21
Kurtosis	−0.12	−0.53	−0.66	−0.76	−0.58	−0.63
Min	0.00	0.00	0.00	0.00	0.00	0.00
Max	156.00	156.00	178.00	155.00	156.00	70.00

**Table 3:** Relationship between BMI (kg/m^2^) and measurement time as well as patients’ sex—data percentage to the year of measurement.

		2016	2017	2018	2019	2020	2021	2022	Total
Women	Underweight	6.1%	6.2%	6.0%	5.8%	5.6%	5.6%	5.4%	5.9%
	Normal body weight	64.4%	63.7%	62.7%	62.0%	61.9%	61.5%	61.3%	62.4%
	Overweight without obesity	19.6%	19.9%	20.7%	20.9%	21.4%	21.6%	22.1%	20.6%
	I degree of obesity	7.1%	7.3%	7.5%	8.0%	8.1%	8.2%	8.1%	7.9%
	II degree of obesity	2.1%	2.2%	2.2%	2.4%	2.1%	2.3%	2.5%	2.4%
	III degree of obesity	0.7%	0.8%	0.8%	0.8%	0.9%	0.8%	0.6%	0.8%
	Total	100.0%	100.0%	100.0%	100.0%	100.0%	100.0%	100.0%	100.0%
Men	Underweight	0.8%	0.8%	0.9%	0.9%	0.8%	0.8%	0.9%	0.8%
	Normal body weight	39.3%	38.9%	38.3%	37.9%	37.1%	37.1%	36.9%	38.1%
	Overweight without obesity	42.7%	42.8%	42.7%	42.4%	42.3%	42.6%	42.6%	42.6%
	I degree of obesity	13.6%	14.0%	14.3%	14.7%	15.3%	15.6%	15.7%	14.6%
	II degree of obesity	2.9%	2.9%	3.2%	3.3%	3.4%	3.2%	3.1%	3.1%
	III degree of obesity	0.6%	0.7%	0.8%	0.8%	1.1%	0.7%	0.8%	0.8%
	Total	100.0%	100.0%	100.0%	100.0%	100.0%	100.0%	100.0%	100.0%

**Table 4:** Association between BMI (kg/m^2^) and age of patients—percentage distribution across age categories.

	<18	18–35	35–54	55–69	>69	Total
Underweight	13.9%	4.9%	1.2%	0.4%	0.4%	3.0%
Normal body weight	61.3%	59.3%	39.9%	26.5%	26.8%	49.3%
Overweight	18.1%	26.0%	40.3%	42.4%	52.1%	32.4%
I degree of obesity	4.2%	7.4%	14.4%	24.4%	18.4%	12.4%
II degree of obesity	2.0%	1.8%	3.3%	6.2%	2.1%	2.6%
III degree of obesity	0.5%	0.6%	0.9%	0.1%	0.2%	0.3%
Total	100.0%	100.0%	100.0%	100.0%	100.0%	100.0%

**Table 5:** Relationship between BMI (kg/m^2^) and age of patients—data percentage to the BMI category.

	Underweight	Normal Body Weight	Overweight without Obesity	I Degree of Obesity	II Degree of Obesity	III Degree of Obesity	Total
<18	0.10%	0.20%	0.10%	0.10%	0.00%	0.00%	0.00%
18–35	83.70%	63.80%	44.30%	33.20%	31.60%	33.80%	54.20%
35–54	14.10%	29.50%	42.10%	49.00%	51.20%	51.60%	38.60%
55–69	2.10%	6.40%	13.40%	17.50%	17.10%	14.50%	7.10%
>69	0.00%	0.10%	0.10%	0.20%	0.10%	0.10%	0.10%
Total	100.00%	100.00%	100.00%	100.00%	100.00%	100.00%	100.00%

**Table 6:** Association between BMI (kg/m^2^) and personal health evaluation—percentage data for health ratings.

	Subjective Health Assessment		Total
	Good	Very Good	
Underweight	3.10%	3.20%	3.10%
Normal body weight	48.10%	58.30%	52.30%
Overweight without obesity	32.10%	31.50%	31.90%
I degree of obesity	14.10%	5.60%	10.30%
II degree of obesity	2.10%	1.10%	1.90%
III degree of obesity	0.50%	0.30%	0.50%
Total	100.00%	100.00%	100.00%

**Table 7:** Association between BMI and the prevalence of specific illnesses—percentage representation for each BMI group.

	Underweight	Normal Body Weight	Overweight without Obesity	I Degree of Obesity	II Degree of Obesity	III Degree of Obesity	Total
Hypertension	29.8%	38.9%	45.7%	50.6%	52.5%	56.1%	45.3%
Type 2 diabetes	8.7%	6.1%	7.1%	10.1%	16.1%	17.1%	8.2%
Lipid disorders	58.7%	52.1%	43.1%	35.5%	28.1%	24.1%	43.5%
Coronary disease	2.8%	2.9%	4.1%	3.8%	3.3%	2.7%	3.0%
Total	100.0%	100.0%	100.0%	100.0%	100.0%	100.0%	100.0%

**Table 8:** Association between BMI (kg/m^2^) and co-existing conditions—percentage data relative to BMI.

	Underweight	Normal Body Weight	Overweight without Obesity	I Degree of Obesity	II Degree of Obesity	III Degree of Obesity	Total
Hypertension	26.4%	31.2%	34.5%	39.4%	42.2%	46.5%	35.1%
Type 2 diabetes	6.8%	3.0%	2.6%	3.4%	4.7%	6.2%	3.6%
Lipid disorders	58.4%	48.1%	32.7%	18.9%	12.3%	8.4%	33.1%
Coronary disease	2.1%	0.8%	1.2%	1.2%	0.8%	0.3%	1.0%
Hypertension + type 2 diabetes	0.8%	0.7%	2.6%	4.4%	8.1%	10.3%	2.7%
Hypertension + lipid disorders	4.3%	12.4%	18.4%	22.3%	18.9%	15.9%	17.6%
Hypertension + coronary disease	0.2%	0.8%	0.5%	1.5%	1.1%	0.9%	0.4%
Type 2 diabetes + lipid disorders	0.3%	0.4%	1.3%	0.9%	1.4%	1.2%	0.8%
Type 2 diabetes + coronary disease	0.1%	0.0%	0.2%	0.1%	0.4%	0.1%	0.1%
Lipid disorders + coronary disease	0.2%	0.8%	0.6%	0.4%	0.4%	0.1%	0.5%
Hypertension + type 2 diabetes + lipid disorders	0.1%	1.2%	2.9%	4.2%	7.6%	7.6%	2.6%
Hypertension + type 2 diabetes + coronary disease	0.1%	0.1%	0.1%	0.1%	0.2%	0.2%	0.1%
Hypertension + lipid disorders + coronary disease	0.1%	0.4%	1.6%	1.9%	0.7%	1.1%	1.6%
Type 2 diabetes + lipid disorders + coronary disease	0.1%	0.0%	0.1%	0.1%	0.1%	0.2%	0.1%
All		0.1%	0.7%	1.2%	1.1%	1.2%	0.7%
Total	100.0%	100.0%	100.0%	100.0%	100.0%	100.0%	100.0%

**Table 9:** Relationship between age and co-morbidities—data percentage to age ^1^.

	Age					Total
	<18	18–35	35–54	55–69	>69	
Hypertension	50.0%	37.50%	34.60%	32.10%	30.50%	34.60%
Type 2 diabetes	50.0%	6.80%	3.30%	2.50%	2.10%	3.40%
Lipid disorders		44.70%	35.10%	18.10%	7.50%	33.20%
Coronary disease		0.40%	0.70%	2.40%	1.70%	0.80%
Hypertension + type 2 diabetes		0.80%	2.20%	4.40%	6.80%	2.40%
Hypertension + lipid disorders		8.50%	18.50%	21.70%	19.40%	17.60%
Hypertension + coronary disease		0.20%	0.30%	2.40%	4.10%	0.70%
Type 2 diabetes + lipid disorders		0.50%	0.80%	1.30%	1.20%	1.20%
Type 2 diabetes + coronary disease		0.00%	0.10%	0.30%	0.70%	0.10%
Lipid disorders + coronary disease		0.20%	0.40%	1.40%	1.40%	0.70%
Hypertension + type 2 diabetes + lipid disorders		0.30%	2.40%	6.30%	7.50%	3.10%
Hypertension + type 2 diabetes + coronary disease		0.00%	0.20%	0.50%	2.10%	0.10%
Hypertension + Lipid disorders + coronary disease		0.10%	1.20%	4.60%	9.50%	1.50%
Type 2 diabetes + lipid disorders + coronary disease		0.00%	0.00%	0.10%	0.60%	0.10%
All		0.00%	0.20%	1.90%	4.90%	0.50%
Total	100.0%	100.0%	100.0%	100.0%	100.0%	100.0%

^1^ Each letter in subscript represents a subset of the age category for which the column proportions do not differ significantly at the level of 5%.

**Figure A1:**
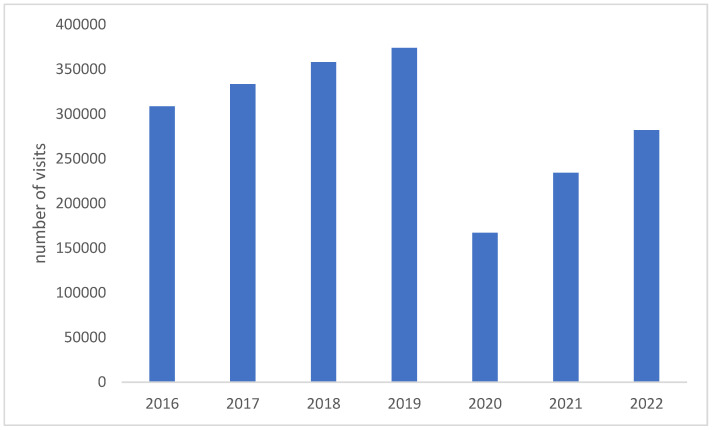
The number of visits analyzed versus the year of measurement.

**Figure A2:**
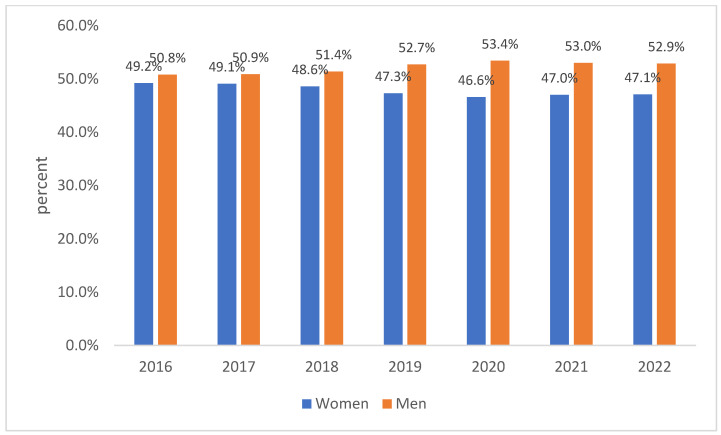
Sex distribution depending on the year of measurement (95% CI: ±0.2%).

**Figure A3:**
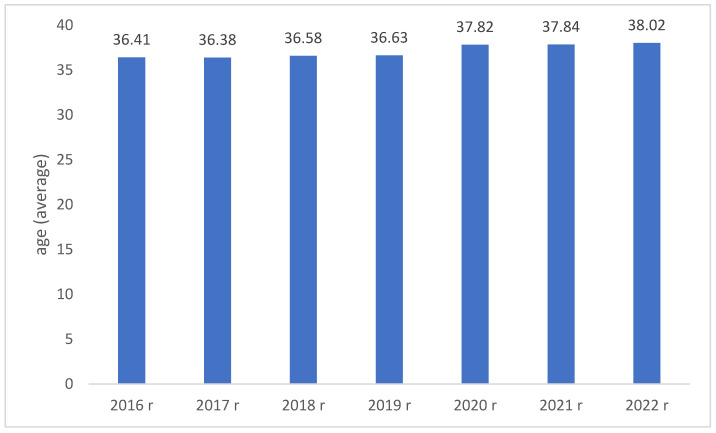
Patient age distribution versus the year of measurement.

## Data Availability

On demand from corresponding author.

## Appendix A. Additional Analyses

This study included 2,056,861 visits to occupational medicine (collected from 1,342,749 unique patients) from 2016–2022. The exact number of collected results depending on the year of measurement is presented below.

Regarding gender, men made up a marginally larger portion at 51.7%. As the study progressed, the proportion of male participants saw a modest rise (refer to Figure A2).

The age span of the participants was from 18 to 90 years, with an average (M) of 36.81 and a standard deviation (SD) of 10.91. There was a subtle pattern indicating that the average age of the subjects studied rose slightly year by year (refer to Figure A3). It is essential to note that as patients age, they naturally move into different age categories. This observation is not just a redundant statement that people age over time.

The detailed breakdown of age groups based on the year of assessment is shown in the following table. It was observed that as the years of measurement progressed, there was a decline in the percentage of individuals aged 18–35, and a rise in the 35–54 age bracket. For other age groups, the trends were not as distinctly noticeable compared to these two age ranges.

**Table A1:** Distribution of age groups versus the year of measurement (with 95% CI).

	2016	2017	2018	2019	2020	2021	2022	Total
<18	0.0% (±0.1%)	0.0% (±0.1%)	0.1% (±0.1%)	0.1% (±0.1%)	0.0% (±0.2%)	0.1% (±0.1%)	0.0% (±0.1%)	0.1% (±0.2%)
18–35	54.5% (±0.2%)	54.3% (±0.3%)	53.3% (±0.2%)	52.5% (±0.2%)	46.6% (±0.2%)	52.1% (±0.2%)	52.3% (±0.2%)	53.3% (±0.2%)
35–54	35.7% (±0.2%)	35.8% (±0.2%)	36.7% (±0.2%)	37.6% (±0.2%)	42.0% (±0.3%)	36.9% (±0.2%)	36.4% (±0.2%)	36.5% (±0.2%)
55–69	9.6% (±0.1%)	9.7% (±0.1%)	9.8% (±0.1%)	9.7% (±0.1%)	11.2% (±0.1%)	10.7% (±0.1%)	10.4% (±0.1%)	9.1% (±0.1%)
>69	0.1% (±0.1%)	0.1% (±0.1%)	0.2% (±0.1%)	0.2% (±0.1%)	0.3% (±0.1%)	0.3% (±0.1%)	0.9% (±0.1%)	0.2% (±0.1%)
Total	100.0%	100.0%	100.0%	100.0%	100.0%	100.0%	0.1% (±0.1%)	100.0%

There were also no significant differences in terms of the distribution of the respondents by year of measurement and the voivodeship of residence (see Table A2).

**Table A2:** Distribution of voivodships depending on the year of measurement (with 95% CI).

	2016	2017	2018	2019	2020	2021	2022	Total
Lower Silesia	12.6% (±0.2%)	13.1% (±0.2%)	12.7% (±0.1%)	13.0% (±0.1%)	13.6% (±0.2%)	12.6% (±0.2%)	11.7 (±0.2%)	12.8% (±0.2%)
Kuyavian-Pomeranian	3.9% (±0.1%)	4.1% (±0.1%)	4.1% (±0.1%)	3.8% (±0.1%)	3.7% (±0.1%)	4.2% (±0.1%)	3.9% (±0.1%)	4.0% (±0.1%)
Lublin	0.9% (±0.1%)	0.9% (±0.1%)	0.9% (±0.1%)	0.8% (±0.1%)	0.7% (±0.1%)	0.9% (±0.1%)	1.1% (±0.1%)	0.8% (±0.1%)
Lubusz	1.4% (±0.1%)	1.4% (±0.1%)	1.5% (±0.1%)	1.6% (±0.1%)	1.9% (±0.1%)	1.4% (±0.1%)	1.5% (±0.1%)	1.4% (±0.1%)
Lodz	7.2% (±0.1%)	7.1% (±0.1%)	6.6% (±0.1%)	6.7% (±0.2%)	6.3% (±0.1%)	7.2% (±0.1%)	7.4% (±0.1%)	7.0% (±0.1%)
Lesser	10.9% (±0.1%)	11.2% (±0.2%)	11.8% (±0.2%)	11.3% (±0.2%)	11.6% (±0.2%)	11.4% (±0.2%)	10.5% (±0.2%)	11.3% (±0.2%)
Mazowieckie	33.6% (±0.2%)	32.0% (±0.3%)	30.8% (±0.3%)	28.6% (±0.2%)	29.0% (±0.2%)	32.0% (±0.3%)	31.6% (±0.3%)	31.1% (±0.2%)
Opole	1.1% (±0.1%)	1.1% (±0.1%)	1.1% (±0.1%)	1.2% (±0.1%)	1.2% (±0.1%)	1.2% (±0.1%)	1.2% (±0.1%)	1.1% (±0.1%)
Subcarpathian	2.0% (±0.1%)	2.4% (±0.1%)	3.4% (±0.1%)	3.1% (±0.1%)	2.8% (±0.1%)	2.4% (±0.1%)	1.8% (±0.1%)	2.8% (±0.1%)
Podlasie	1.8% (±0.1%)	1.9% (±0.1%)	1.7% (±0.1%)	1.6% (±0.1%)	1.5% (±0.1%)	1.9% (±0.1%)	1.4% (±0.1%)	1.7% (±0.1%)
Pomeranian	6.2% (±0.1%)	6.3% (±0.1%)	6.8% (±0.2%)	7.3% (±0.1%)	6.5% (±0.1%)	6.3% (±0.1%)	6.5% (±0.1%)	6.7% (±0.1%)
Silesian	6.1% (±0.1%)	6.2% (±0.1%)	6.2% (±0.1%)	8.2% (±0.1%)	8.3% (±0.1%)	6.2% (±0.1%)	5.6% (±0.1%)	6.8% (±0.1%)
Świetokrzyskie	0.7% (±0.1%)	0.7% (±0.1%)	0.7% (±0.1%)	0.7% (±0.1%)	0.7% (±0.1%)	0.7% (±0.1%)	0.8% (±0.1%)	0.7% (±0.1%)
Warmia-Masurian	2.1% (±0.1%)	1.9% (±0.1%)	2.0% (±0.1%)	2.1% (±0.1%)	2.1% (±0.1%)	1.9% (±0.1%)	2.2% (±0.1%)	2.0% (±0.1%)
Greater	7.0% (±0.2%)	6.8% (±0.2%)	6.7% (±0.3%)	6.4% (±0.2%)	6.7% (±0.2%)	6.8% (±0.2%)	7.5% (±0.2%)	6.7% (±0.2%)
West Pomeranian	2.6% (±0.1%)	3.0% (±0.1%)	3.0% (±0.1%)	3.5% (±0.1%)	3.5% (±0.1%)	3.0% (±0.1%)	5.3% (±0.1%)	3.1% (±0.1%)
Total	100.0%	100.0%	100.0%	100.0%	100.0%	100.0%	100.0%	100.0%
